# Inclusion at universities: Psychometric properties of an inclusive management scale as perceived by students

**DOI:** 10.1371/journal.pone.0262011

**Published:** 2022-01-07

**Authors:** María José Solis-Grant, Camila Espinoza-Parçet, Cristóbal Sepúlveda-Carrasco, Cristhian Pérez-Villalobos, Iván Rodríguez-Núñez, Cristian Pincheira-Martínez, Juan Pablo Gómez-Varela, Daniela Aránguiz-Ibarra

**Affiliations:** 1 Kinesiology Department, School of Medicine, Universidad de Concepción, Concepción, Chile; 2 Universidad de Concepción, Concepción, Chile; 3 Universidad de las Américas, Santiago, Chile; 4 Universidad Santo Tomás, Santiago, Chile; 5 Universidad San Sebastián, Santiago, Chile; Aalborg University, DENMARK

## Abstract

**Introduction:**

During the last century, the inclusion of all kinds of diversity became a social imperative in all social spaces but above all in some institutions such as the educational ones. Among these, inclusion has been least studied in the tertiary education organizations. This communication proposes and evaluates the psychometric properties of a new instrument, named Inclusive Management in Tertiary Institutions Scale (IMTIS), to assess inclusive management in universities.

**Method:**

The researchers used a quantitative research model through survey. We based on the Index for Inclusion to design the IMTIS. We first submitted it to the assessment of experts. Then we applied the resulting version in an online survey including a sample of 1557 students from two universities and 121 different undergraduate careers. A panel of experts judged the content validity of the instrument. Participants answered the IMTIS after informed consent. We used confirmatory factor analysis to assess the construct validity of the instrument. We also evaluated the reliability of the measurements.

**Results:**

From a kit of 33 originally proposed items, we obtained a version of 22 items with CVR between 0.60 and 1.00, and a IVC = 0.78. The confirmatory factor analysis showed that the six-factor solution had a better adjustment than the one and three factors solutions (RMSEA = 0.059; CFI = 0.947; TLI = 0.937). The McDonald ω coefficients were between 0.864 and 0.922.

**Conclusion:**

The results deliver evidence that supports the validity and reliability of the IMTIS measurements to carry out research and diagnosis of inclusive management in higher education institutions.

## Introduction

The inclusion of diversity in the educational system represents a set of social conceptions oriented to the elimination of barriers to learning and promote the participation of the whole student body, value the diversity of each person and achieve a feeling of belonging in everyone [1–3]. The right to quality education is recognized, as well as its close relationship to justice and equity that underlie the inclusive education concept [1, 4, 5]. This, because inclusion, as we understand it, is a dynamic process, changeable, rich, and attainable that is interconnected with the educational concepts of values, policies, and social justice practices [6].

The recognition of inclusive education based on equity and social justice for everyone has its origin in the Universal Declaration of Human Rights [7]. These principles have been progressively and universally assumed in forums and international declarations, among them the World Conference on Education for All, held in Thailand in March 1990, where three fundamental values were proposed: acceptance, belonging, and community feeling [8]. Some years later, UNESCO, together with the Ministry of Education and Science of Spain, convoked a World Conference held in Salamanca, to strengthen the engagement about the right to quality education for all persons and specifically for those having special educational needs [9]. Later on, the initiative “Education for all” adopted in the World Education Forum held in Dakar projected an international scale engagement to attain quality education without exclusions [10].

Beyond the purely educational framework, the role of the International Convention on the Rights of People with Disabilities must also be highlighted. Its purpose is to promote, protect and assure all the human rights and fundamental freedom for people with disabilities. In it, the countries engage themselves to guarantee an inclusive educational system at all levels, so as to make this right effective without discrimination and on the basis of equal opportunities [11]. Along these lines, various international organisms have encouraged the countries to adopt constant processes to promote inclusive education, not only focusing on the field of disability but from a broad perspective of giving value to the diversity inherent in human beings, including the latest UNESCO Global Monitoring Report on Education worldwide [12] and the 2030 agenda for sustainable development that proposes as one of its objectives quality inclusive education, promoting lifelong learning opportunities for all, including equal access to technical and vocational education and training [13].

The progress of education towards a more inclusive perspective has been strongly determined by the recognition of the rights of disabled persons, triggered by the demands of that group [14, 15]. However, diversity must be considered from multiple perspectives and not only the condition of disability; it is necessary to pay attention to the cultural, ideological, religious, racial and political spheres [16]. Indeed, currently, those studies that deal with the field of disability declare an evolution of their conceptual paradigms, from "disability policies" to "inclusion policies" [17, 18]. This is also shown in various guidelines of entities such as UNESCO and OECD [18], that seek to advocate the elimination of excluding procedures, shown as specific attitudes and answers towards diversity and to certain dimensions of the human being related to vulnerability, economic and/or cultural disadvantage, ethnic diversity, disabled situations, religion, sex, gender and sexuality [2, 17, 19].

From these concepts, Inclusion answers to social transformations that affect the way in which the awareness of difference is addressed and resolved. Therefore, educational inclusion would be constituted by four fundamental characteristics: it is a process, it is centered on the identification and elimination of barriers, supposes the presence, participation and progress of the whole student body, paying special attention to groups of students at risk of marginalization or exclusion [2].

In different countries, educational inclusion has become part of the more relevant public demands of social groups that have been traditionally been postponed from higher education [5, 14–16, 19–21].

In Chile, this has been translated into the implementation of laws such as the higher education [22], the Law establishing measures against arbitrary discrimination [23] and the law about equal opportunity and social inclusion of disabled persons [24], where a just and quality education is promoted, an education that advocates the elimination of all forms of arbitrary discrimination, democratization of education, adaptation of materials and implementation of methodological strategies to include everyone [22–24]. In spite of the efforts to attain inclusive education, it is still estimated that 258 million children, adolescents and young people do not enter the school system and represent 17% of the world’s population. Specifically, in higher education the percentage is even higher, reaching approximately 60% of people that do not enter that educational level. Therefore, the gross average world enrollment rate reached only 38% in 2018. Furthermore, wide differences were observed between low-income countries with a 9% enrollment, as compared to high income countries, where a 75% was attained [12]. Similar percentages have been observed in Chile, through data of the Ministry of Education that reveal that around 60% people do not accede to higher education institutions [25]. In this challenging context, to promote inclusive education has become a priority objective for the present society on a world level [26].

Within the responses that higher education institutions develop in much of the world to address student diversity and inclusion, these have been driven by the rhetoric of equity and social justice [27]. This is why the concept of inclusion in education, defined as a means to achieve equitable access of the diversity of students to quality education, takes on special importance, without discrimination [28]; a scenario in which a link between the inclusion of diverse students and quality in higher education is required [3, 29–32], because equity is closely related to the inclusion of diversity, and is considered a central element in the assurance of educational quality [30].

In Latin America, the incorporation of policies and programmes in higher education that are in favour of social inclusion and equity have posed challenges similar to those observed in other areas of development, since this region still has deep social inequalities [33, 34]. This is due to the current student body in higher education which is more diverse and complex, leaving behind its composition, which was once exclusive to the elites, and nowadays its students come from diverse social and cultural backgrounds [35]. Thus, it is perceived that a new approach is needed in policies and practices that must be accompanied by attitudinal changes and the learning of strategies that promote inclusive environments, where the value of each individual is most important, including those characteristics different from their own, such as their way of communicating, their ethnicity or creed [36, 37]. It is also essential to consider the social benefits and other dimensions to which higher education contributes, understanding it from a broad perspective for the development of learning, which impacts not only on the individual level, but on society as a whole and, of course, on those social groups previously underrepresented in higher education and which currently have greater access to this educational level. Therefore, higher education must not be reduced to its economic value in the markets or quasi-markets [36]. For many inclusion practitioners, higher education institutions incorporate the concept of ’diversity in their discourses to increase the market value of their institution. However, when such practitioners attempt to incorporate diversity into the institutional culture, they are confronted with a ’brick wall’ [38]. Moreover, the positive impact of diversity on student learning tends to be underestimated [29].

The democratically based discourses, which call for the inclusion of student diversity in university education, allow for the generation of dialogues that lead to institutional participation, in this way, the "positive affective value" of diversity becomes a useful tool for professionals who manage inclusion programmes, managing to chip away at this "brick wall" of the academy [38]. From this perspective, recent literature points that in Spanish universities there is a medium level of institutionalisation of diversity [27] or even a disconnection between institutional regulations and their practical implementation in other Ibero-American contexts [32]. This is because universities are not stable, but are in a continuous process of being "instituted" through practices such as recruitment and employment [38], seeking a benign commitment to diversity that often fails to achieve fundamental changes in the distributions of power, resources and opportunities, but only intensifies existing inequalities. Therefore, such organisational commitments to multiculturalism and diversity often fall short in practice [39]. This is how through this continuous process of assemblage and reproduction, whiteness becomes the norm; thus, the white body prevails in the university system, while the minority body is always an outsider and marked as other [38]. This manifests itself in multiple facets of society such as gender, race, ethnicities, sexual orientation, disabilities and many others [19, 29, 37, 40, 41].

In the United States and South Africa, diversity regulations in universities focus primarily on issues of disability, race and ethnicity, responding mainly to existing legislation [41]. This response to legislation has also been observed in other countries such as Australia and the United Kingdom, where diversity has been "institutionalised" with the incorporation of this concept in the institutional mission and policies, as well as the creation of programmes and the hiring of professionals in the area of inclusión. However, all these actions do not necessarily entail a genuine commitment by the institutions to diversity and inclusion [38]. This view and the tensions that are generated from it are consistent with what Thomas (2018) proposed on "Diversity Regimes” [39]; on the one hand, the imperative need to adopt a perspective focused on the generation of social value and an inclusive approach linked to quality is disseminated and highlighted [42–45]; on the other hand, the concept of quality has been assumed and measured in terms of productivity and efficiency in a market-based context, under which there is no room for the valuation of diversity and the social role [27], marginalising non-monetary values with the strengthening of competence mechanisms centred on the hegemony of rankings as surveillance and control procedures [46].

Considering that educational inclusion in higher education is a recent field of study and a very important area to be taken into account in universities [35, 37], it seems necessary for educational institutions to assume a commitment and manage coherently the promotion of accessibility and inclusion for all as an institutional premise. Only in this way can inclusive education be successfully achieved [47]. Therefore, the management of organizational behavior should guide the beginning of the change, involving both the individual level as all the processes of the institution, assuring an adequate interaction and connection among the administrative guidelines, the campus, the culture and the specific aspects to be implemented in the classrooms in order to obtain results that significantly promoted social justice [3, 48]. To this end, the establishment of multidimensional self-assessment processes about inclusion in educational institutions, as well as the generation of research lines in the area, are essential to attain the objectives of an inclusive sense and social justice [49–51]. In this context, the creation of instruments that assess this type of aspects is essential [52].

The inclusive education guide “*Index for inclusion*” (Index), constitutes a self-assessment instrument for school institutions in an inclusive education framework. Three dimensions are proposed in it: culture, policies and inclusion practices [2]. These dimensions have been established as a valuable tool that reveals that the difficulties for learning and participation experimented by many students are mainly related to the excluding configuration of the cultures, policies and practices of the educational community rather than to their personal characteristics [5].

The Index implements its three dimensions using six domains, guided by inclusive values, to develop progress and innovation processes in the schools [2]. These domains are: 1) Build a community; 2) Establish inclusive values within the inclusive culture dimensions; 3) Develop a school center for all; 4) Organize support to diversity within the inclusive policy; 5) Build a curriculum for all, and 6) Orchestrate learning within the Inclusive Practices dimension.

The Index proposal was originally designed for British school centers, however in time it has been adapted in numerous countries and has been translated to 37 languages. The index is still applicable today, with its third edition published in 2011 and subsequently adapted and translated into Spanish in 2015 [2]. Furthermore, some research that use the Index domains have appeared, adapting them for application in higher education [53, 54].

During 2015, a preliminary study for the adaptation of the Index in universities was published in Spain. This version of the questionnaire was submitted to the judgment of experts, but there was no significant agreement in the evaluations delivered by the specialists; it is recommended that future studies should rephrase some of its indicators and advance in the empiric procedure, so as to count on an instrument that adapts the Index to the university context [53].

On the other hand, a 2016 study that analyzed instruments for inclusive education at the school level, concluded that there was a large number of instruments that have been prepared and exclusively directed to obtain the opinion of the teachers. In this context it is recommended to increase the research universe, leading to reflect the opinions and diversity of the student body, and effective documents are required to this end, to incorporate the feelings of this group as well as of the total number of persons that conform the educational community [52].

Specifically, at the university education level, it is recommended to use fast application methodologies, valid and trustworthy, to assess inclusive environments and enquire how well is the inclusion understood by the main agents of the educational community: direction, teachers and students [48, 55]. This taking into account that it is still necessary to consolidate policies that promote inclusion and value persons’ diversity at this educational level [17].

Taking into account the mentioned antecedents, this research seeks to propose a new instrument called Inclusive Management in Tertiary Institutions Scale (IMTIS), (in Spanish *Escala de gestión inclusiva en Instituciones Terciarias*), to assess inclusive management in universities, following the six domains proposed by the Index.

The instrument was designed to be applied to students, teachers, non-teaching personnel, and administration with the purpose of allowing an inclusive and participative assessments, incorporating all the actors of the educational community. In this instance, this study seeks to offer initial evidence of its psychometric properties in undergraduate students this time, considering evidences of content validity, construct, and reliability. We used a sample of undergraduate students this time, given that the Index for inclusion highlights the leading role of this group when it comes to an understanding of the processes of inclusion and exclusion in the institutions. Also, they are the students the first whom we seek to impact with the actions of inclusive management. Therefore, they are key agents expressing how these management elements are perceived. They can provide new reflections on these processes more frequently and give the possibilities of inclusion that can initiate in the educational institutions [1, 2].

## Method

We performed a quantitative, non-experimental, transversal and analytical study.

### Participants

The population was made up of two Chilean universities’ undergraduate students, one private and the other public. We obtained a sample of 1557 students (S1 Table) from 121 different undergraduate careers employing a non-probabilistic sampling by convenience. Of these, 1,067 (68.53%) were women, 455 (29.22%) men, and 35 answered the “Other” option in the gender question (2.25%). Their ages varied between 18 and 66 years (M = 25.26; SD = 7.45).

### Data collection

The students answered the Inclusive Management in Tertiary Institutions Scale (IMTIS), prepared by the research team and based on the Index for Inclusion of Booth & Ainscow.

The instrument showed a set of items describing expected inclusive practices in a higher education institution (university, professional institute, or technical training center), based on the six domains of the Index: Build a collaborative community, Promote inclusive values, Develop a university for everyone, Organize support to care for diversity, Manage the educational process and Mobilize resources, these are in turn organized in three dimensions.

The Index proposes a series of questions in each of its six domains to address in school centers and to conceptualize there. However, in this study, a theoretical definition proposal was integrated into each of the six domains so as to simplify their understanding and scope. This conceptualization was done by the research team, starting from the definitions revised in the literature [2, 53, 54] and applying it to the specificities of higher education. In this way, inclusion was conceptualized as a process related to the participation of all persons in the creation of participation systems and their adjustment, the promotion of inclusive values, institutional culture, educational community and curriculum, decreasing all forms of exclusion and discrimination [2]. Therefore, all persons and all forms of diversity are considered for this process, including, although not limited to, gender, sexual orientation, inter-cultural, disabled condition, socioeconomic, religion, etc. [2, 17, 19].

The definitions employed in the study are shown in Table 1.

**Table 1:** Index Inclusion domains and definitions proposed for higher education.

Domain	Definition
Build a collaborative community,	Frequency in which the actions on the part of the members of the community contribute to that everyone should feel part of it, identifying it as a safe, friendly, collaborative and stimulating environment, where diversity is valued and each person has the possibility to attain the highest levels of attainment
Promote inclusive values	Frequency in which actions are effected on the part of the institution to transmit am inclusive philosophy to all members of the community
Develop an educational institution for everyone	Frequency in which the institution formally fosters, from its normative framework, the generation of actions so that all members of the higher education institution, without distinction, may develop their potentialities.
Organize support to care for diversity,	Frequency in which the institution implements effective strategies, adopted by all the different levels of the educational institutions to answer to the diversity needs of its members.
Manage the educational process	Frequency in which the formative process within the institution are intentionally carried out in an inclusive manner, deliberatively allowing that all students should have Access to learning opportunities and avoiding the apparition of barriers and discriminatory practices.
Mobilize resources	Frequency in which the internal and external resources (financial, human and material) are known, distributed and used to support the learning and participation of the students of the institutions, independently from their diversity.

Starting from these definitions, we operationalized the IMTIS items for higher education by formulating a set of items that indicated expected inclusive practices in a higher education institution, following the six domains of the Index.

In order to allow assessments that would incorporate the different actors of the educational community, we worded the items of the first four factors that allude to the whole institution to apply both to undergraduate and postgraduate students, teacher, non-teaching staff, managers, and directors. As for the last two factors, which specifically refer to the formation process, we worded them to be answered by teachers and students only.

To answer, the participants had to reply with what frequency did they identify the happening of each of these practices in their educational institution, using a scale of seven options: (0 = Never, 1 = Almost never, 2 = Rarely, 3 = Sometimes, 4 = Frequently 5 = Almost always, and 6 = Always).

The original proposal of the instrument had 33 items, but after the experts’ revision, there were 22 items left.

### Procedure

Our research team designed the IMTIS, which was formed by an inter-professional panel of experts in educational inclusion, higher education, and psychometrics from four universities, two professional centers, and a technical formation center, all from the higher education system of Chile.

This research team prepared an initial proposal of 33 items, and we submitted it to the expert’s judgment so as to assess its content validity. Then, 20 professionals from higher education institutions (17 of Chile, two from Spain, and one from Ecuador), specialists of educational inclusion in general or some specific diversity, assessed the items as regards their theoretical congruence with the dimension sought to be measured.

Then, we obtained a final version of 22 items using the experts’ judgment results, which we applied to the sample of 1557 undergraduate students. In the first place, we asked for institutional authorization from the participating universities to carry out this survey. After we obtained it, the universities should send an official e-mail to the students, inviting them to participate in the survey. This mail contained a hyperlink to access the questionnaire in the survey online platform SurveyMonkey©. Those who entered the survey form had first to read the informed consent, where we explained the purpose of the study, methodology aspects, requested participation, associated guarantees such as anonymity, free and voluntary participation, and the right to withdraw at any time without consequences. The survey showed two options after the informed consent: “I accept to participate according to the established conditions” and “I do not accept to participate under the established conditions”. Only those who accepted the informed consent would continue the survey. The page closed for those that did not accept.

### Ethics

The study received the approval of the Ethics, Bioethics and Biosafety Committee of the Research and Development Vice rectorate of Universidad de Concepción, Chile, the institution responsible for the project (resolution number CEBB 703–2020), and of the Science Ethics Committee of the Research Vice rectorate of Universidad de las Américas (resolution number CEC_FP_2021–005).

### Data analysis

To obtain evidence about the scale’s content validity, we analyzed the experts’ judgment calculating the Coefficient Validity Ratio (CVR) of the judge’s assessments concerning the item’s relevance to measure its respective domain. Later on, we calculated the Content Validity Index (CVI) to estimate the instrument’s validity as a whole global content validity of the instrument as a whole.

We applied the IMTIS to the students’ sample, using the final version of the scale resulting from the previous analysis. Its resulting data was analyzed using a Confirmatory Factor Analysis (CFA) in order to confirm the scales’ internal structure. We compared fit indexes for 6-factors, 3-factor, and one-factor models. And finally, we also compare them to a bifactor and a second-order factor model. We evaluated the models fit using χ^2^-test, but due to its power and its tendency to reject slightly miss-specified models [56, 57], we complemented it by other four indices: Comparative Fit Index (CFI), Tucker-Lewis Index (TLI), Root Mean Square Error of Approximation (RMSEA) and a 90% confidence interval, and Standardized Root Mean-Square (SRMR) [58]. We used as cut-off values for an acceptable goodness of fit the following: CFI > 0.95, TLI > 0.95, RMSEA < 0.06, and SRMR < 0.08 [59, 60].

We employed Mplus 8.4 to conduct CFA. Due to the obtained Mardia’s coefficient kurtosis was 734.136, we decided to conduct it using the Robust maximum likelihood estimator.

We evaluated internal consistency using Cronbach’s alpha and Omega reliability coefficients. Cronbach’s alpha is the most widely use reliability coefficients [61], but Omega provides a less biased estimation of it [62].

## Results

### Content validity

The analysis of the values assigned by 20 experts to each dimension by means of the calculation of the CVR index by item and dimension and of the IVC for the whole scale, allowed to envision the items that showed lesser CVRs (See Table 2). In this way six items were eliminated from the 33 proposed items, as they showed CVRs equal to or lower than 0.5, which is the cut-off value proposed by Lawshe [63] for 20 evaluators. In the same manner -as product of the qualitative analysis of the assessors’ comments- it was decided to eliminate another five ítems that showed a CVR = 0.60, significant queries about their content or duplication of contents already assessed in items with a better CVR.

**Table 2:** IVC and CVR indexes obtained for the original and final scales.

Dimension	Initial Number	Essential	Initial CVR	Selected item	Final number
Build a collaborative community	**1**	14	0,40	Eliminated due CVR	
	**2**	19	0,90	Selected	15
	**3**	16	0,60	Selected	10
	**4**	17	0,70	Selected	11
	**5**	15	0,50	Eliminated due CVR	
	**6**	15	0,50	Eliminated due CVR	
Promote inclusive values	**7**	17	0,70	Selected	9
	**8**	18	0,80	Selected	2
	**9**	16	0,60	Eliminated due remarks	
	**10**	20	1,00	Selected	4
	**11**	16	0,60	Eliminated due remarks	
Develop an educational institution for all persons	**12**	17	0,70	Selected	13
	**13**	17	0,70	Selected	1
	**14**	12	0,20	Eliminated due CVR	
	**15**	15	0,50	Eliminated due CVR	
	**16**	17	0,70	Selected	14
	**17**	17	0,70	Selected	12
	**18**	18	0,80	Selected	5
Organize support to attend to diversity	**19**	18	0,80	Selected	8
	**20**	20	1,00	Selected	7
	**21**	18	0,80	Selected	3
	**22**	16	0,60	Eliminated due remarks	
	**23**	15	0,70	Selected	6
Manage an inclusive educational process	**24**	18	0,80	Selected	16
	**25**	20	1,00	Selected	21
	**26**	18	0,80	Selected	19
	**27**	16	0,60	Eliminated due remarks	
	**28**	18	0,80	Selected	20
Mobilize resources for inclusive formation	**29**	16	0,60	Selected	22
	**30**	16	0,60	Eliminated due remarks	
	**31**	14	0,40	Eliminated by RVC	
	**32**	18	0,80	Selected	17
	**33**	18	0,80	Selected	18
		**IVC Scale**	**0,68**		

In this way an average CVR of each dimension, fluctuating between 0.72 and 0.85 was calculated using the selected 22 items and obtaining an IVC of 0.78.

### Construct validity: Confirmatory factor analysis

**Fig 1:**
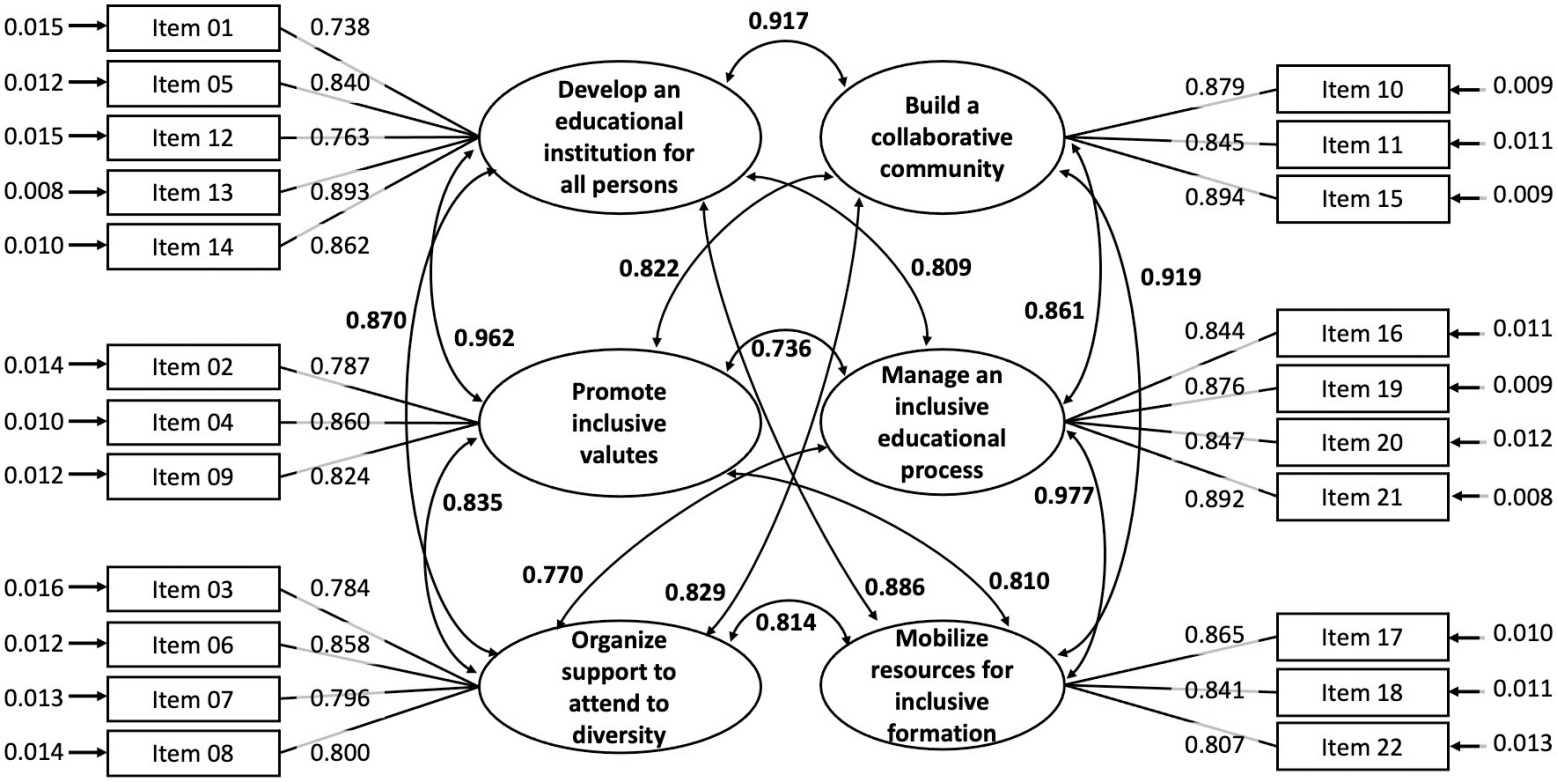
Confirmatory factor analysis of a 6-factors model for the inclusive management in tertiary institutions scale.

The Index assesses inclusive management in higher education taking into account six domains which are in turn collected in three dimensions [2], all forming a single construct which in fact has been empirically supported by factorial analyses [64]. For this reason, the solutions of one, three and six factors seemed as theoretically likely, although the instrument had been designed taking the last one into account. Therefore, we evaluated the goodness of fit for each of these three solutions. The six-factor model showed the highest goodness of fit among the three models. It also showed a good fit for three of the four fit indexes (Fig 1). Only the TLI was under but close to the cut-off point (Table 3). Also, all standardized factor loadings in it were over 0.70 and significant [65]. But the obtained factors also showed substantially high correlations between each other (between 0.736 and 0.977) (Table 4).

**Table 3:** Fit indexes of models 6-factor, 3-factor, and One-factor models and Bifactor and higher-order models for the inclusive management scale as perceived by students.

	gl	χ^2^	RMSEA (IC 95%)	SRMR	CFI	TLI
6-factor model	194	1 226.339*	0.059 (0.056–0.062)	0.034	*0*.*947*	*0*.*937*
3-factor model	206	1 777.912*	0.071 (0.068–0.074)	0.041	*0*.*919*	*0*.*910*
One-factor model	210	3 145.747*	0.096 (0.093–0.099)	0.061	*0*.*850*	*0*.*834*
Bifactor model	188	1 499.156*	0.068 (0.064–0.071)	0.048	0.933	0.917
Higher order model	203	1 772.800*	0.071 (0.068–0.074)	0.046	0.920	0.908

Note:

* denotes a p-value <0.001;

n.s. denotes a non-significant χ2.

**Table 4:** Correlations between six factors of inclusive management scale as perceived by students.

	*1*	*2*	*3*	*4*	*5*	*6*
1. Develop an educational institution for all persons	-					
2. Promote inclusive values	*0*.962*	-				
3. Organize support to attend to diversity	*0*.870*	*0*.835*	-			
4. Build a collaborative community	*0*.917*	*0*.822*	*0*.829*	-		
5. Manage an inclusive educational process	*0*.809*	*0*.736*	*0*.770*	*0*.861*	-	
6. Mobilize resources for inclusive formation	*0*.886*	*0*.810*	*0*.814*	*0*.919*	*0*.977*	-

Note:

* denotes a p-value <0.001

Due to these high correlations, we also performed two alternative solutions: a) a Bifactor Model where the 22 items loaded on the six factors proposed by the *Index for inclusion*, and also on a general factor, and b) Higher-Order Confirmatory Factor Analysis, where the 22 items loaded on the six factors and these six factors in turn loaded on a second-order factor. We also informed the results of these last analyses at the end of Table 3. Bifactor and Higher-Order models showed only acceptable SRMR values. Although they showed better fit than one-factor and 3-factor models, they were under the fit achieved by the 6-factors model, which remains the best between the evaluated solutions.

Therefore, it was decided to accept the six factors solutions due to its better adjustment fitness, in spite of the high correlations between the factors.

### Reliability: Internal consistency

In order to assess the internal consistency of the identified factors, Cronbach’s α reliability coefficient was used: it is usually employed to these ends [61]. The McDonald ω coefficient was also calculated: it would deliver a less skewed estimate of reliability [62]. Table 5 results show that the results of both coefficients are similar, differing in the third decimal place only. They fluctuate between 0.86 and 0.92 in both cases.

**Table 5:** Correlations between six factors of the inclusive management scale as perceived by students.

	α	ω
Develop an educational institution for all persons	*0*.*910*	*0*.*912*
Promote inclusive values	*0*.*864*	*0*.*864*
Organize support to attend to diversity	*0*.*883*	*0*.*884*
Build a collaborative community	*0*.*907*	*0*.*906*
Manage an inclusive educational process	*0*.*920*	*0*.*922*
Mobilize resources for inclusive formation	*0*.*878*	*0*.*876*

## Discussion

The purpose of this study was to propose an instrument to assess inclusive management in higher education institutions. The Inclusive Management in Tertiary Institutions Scale (IMTIS) is different from those previously developed, since most are oriented to the school context characteristics [1, 2, 49, 52, 64, 66, 67], and mainly centered on the formative processes within the classroom [55] or lack sufficient evidence to support their validity [53, 66]. For this reason, the IMTIS answers to the need to count on a tool that would allow continuous diagnosis and assessment of the inclusive practices of the higher education organizations management, as this educational level has specific characteristics differing from school establishments.

On the one hand, in Latin America the guidelines related to inclusive policies in higher education are more recent than those developed for school education and they do not always translate into educational practices [15]. On the other hand, a greater diversity of higher education students has become evident, at present they come from diverse social and cultural sectors [35], also including foreign students. This trend has become stronger in European countries during the last years [19], then, the territory from which the students come is evidently rather more extensive and varied, when compared to what happens in school education, since in the latter most of the students are from the geographical location where the school is sited. Thus, the outreach and society relations activities also occupy a larger territory and play a leading role in higher education, clearly influencing the countries social and economic development of them [68].

We built IMTIS taking into account six domains that assess inclusive management. These domains stem from the three dimensions of the *Index for inclusion* that has been successfully applied in school education institutions [1, 2], and whose fields have been incorporated -in a greater or lesser measure- during the last years in diverse proposals to assess the inclusion and diversity in higher education institutions [51, 53–55]. However, it was still required to consolidate an adaptation of the Index dimensions for this educational level: this is necessary and possible [53].

Although Salceda and Ibáñez published a Spanish adaptation for higher education in 2015, it was a preliminary study. It was fundamentally set in the theoretical reflection plane and ended with an experts’ validation and the proposal of an initial version of a questionnaire. But its results were not sufficient to sustain its validity [53].

When assessing an instrument’s psychometric properties, the first concern to be resolved is that of its content validity. This evaluates how the items achieve a theoretically coherent and conceptually exhaustive coverage of the assessed phenomenon [69]. This assessment allowed to consolidate 22 items belonging to the dimensions and showing adequate content validity.

Regarding the construct validity assessment, we employed confirmatory factor analysis to assess the scale’s internal dimensions. Since we developed the IMTIS on the basis of the Index, which proposes three dimensions and six domains, the proposal for this scale submitted to experts’ judgment suggested six factors theoretically consistent with the Index but conceptually adapted to the Chilean university reality. However, Fernández-Archilla and his colleagues [64] obtained only one factor in the factor analysis they performed in their questionnaire. Therefore, there were three plausible solutions, of six, three, and only one factor. In this regard, the IMTIS showed that the six factors solution–from which the scale was built- showed higher goodness of fit than the three and the one-factor solutions.

Due to the high correlation between the six factors, a Bifactor model and a Higher-order model were also plausible. For the bifactor model, we proposed that 22 items loaded in the previously established six factors and on a general factor as well. For the Higher-order model, we presented that the 22 items loaded on the same six factors and these six factors in turn loaded on a second-order factor. But, these solutions showed lower fit indexes than the 6-factors solution too.

So, we decided to support the 6-factors model that showed an RMSEA and an SRMR with adequate adjustments, and CFI and TLI with acceptable values near the cut-off point, supporting the construct validity for the measure of six factors.

The IMTIS differs from the Fernández-Archilla and his colleagues proposal regarding the education level in which the study was made, in school education and the present in higher education and regarding the number of items and factor, the authors obtained 38 items and a single factor that grouped all the dimensions of the Index [64].

So, in the Index, the first domain is called “Building a community,” and in IMTIS, we called it “Building a collaborative community.” In this manner, it seeks to add an active and participatory connotation on the part of all educational community members, considering that students are adults who contribute horizontally to the development of the institutional culture. Thus, the domain incorporates the idea of collaboration, which provides an added value to this domain, specifying that the inclusive culture could consolidate only if the community members contribute and feel part of it.

The second factor found in this study, deriving from the Index domain “Establishing inclusive values”, is now conceptualized as “Promote inclusive values”, taking into account that the universities must still advance in the institutionalization of matters of inclusion and diversity, developing social justice promotion actions [51]. Furthermore, this aspect must pervade s society in general, contributing to the collective construction of a more included and inclusive citizenship [53], and it must be taken into account here that there are differences between the community role of the schools and the one assumed by a University since it is more localized for the first and differs from the extension and scope of a higher education institution. When referring to the Index domain named: “Developing a school center for everyone”, it is evident that the concepts employed must be adapted to higher education, for this reason, in the case of the third factor found for the IMTIS, we used the Salceda and Ibáñez proposal: “Develop an educational institution for all persons” [53] which is centered on the actions that the institution formally fosters from its normative framework so that all persons, without exception, may develop their potentialities. When this is carried to the inclusive education framework, it translates into offering all persons’ attention in answers to their diversities [70].

The Index concept for the fourth factor named: “Organize support to attend to diversity” is retained. However, it is important to highlight that in higher education, the existence of an extensive institutional norms framework is of greater importance, and it must consider inclusion as a principle able to transcend into all the policies and educational practices [54]. Therefore, the organization of the supports must be contained into this norms framework, and through it, effective and transversal strategies must be implemented to respond to the diverse needs of the persons in the educational community.

For the two last factors found in this study, corresponding to the dimension “Inclusive practices”, the proposals of adaptation of the Index to higher education of Salceda and Ibáñez were taken into account. The authors define them as “Orchestrate the educational process” and “Mobilize resources”, and in them they propose an integration of teaching with the support networks to overcome barriers to learning and participation [53]. Only a terminology change was made in this study to ease the understanding of the concept, so the fifth factor found is named: “Management of the educational process”, and the last factor remained as “Mobilize resources”.

Here, it is essential to point out that the viewpoint of the different higher education policies cannot be reduced to their economic value in the market since the development of learning must necessarily encompass a wider dimension that includes social aspects translated into a true contribution of higher education, to societies, groups, and individuals [36]. In this way, it must be a space for reflection with a strong social compromise towards the internal and external community, the valuation of the inherent diversity of human beings attaining special relevance and the transmission of those values through an inclusive institutional philosophy to reach all persons. Therefore, for these two last domains, inclusion constitutes a transversal axis both for the institution’s formation processes as for the management of resources to support learning and the financial, human, and material participation.

Finally, the internal consistency of the six identified factors’ measurements fluctuated between good (>0.8) and excellent (<0.9) according to the ranges proposed by George and Mallery [71], and they would be suitable for use in research and diagnosis activities [61]. Although there are no interpretation ranges for the McDonald omega coefficient, it is proposed that this would be near 1, so the values obtained in this study would indicate high reliability, which complies with this scenario. Furthermore, the differences between these two coefficients are found in the third decimal point only.

So, the measurements’ internal consistency presents an adequate precision to be used in research and diagnostics, allowing to carry out a precise assessment of the six domains of inclusive management in higher education institutions.

## Limitations

One of the limitations is the type of sampling employed by volunteers with a non-probabilistic selection of participants. In spite of this, it was possible to gather a sample of 1,557 study subjects, representing 121 different undergraduate programs of two administratively different universities.

It is important to note that the universities included in the research are functionally different in the context of the Chilean educational system and include different communes of the country. It is desirable that it would be possible to extend the study to more institutions with the purpose of widening the geographical area and arrive at a population that reflects the diversity existing in the rest of the national territory, Latin America, and other Spanish-speaking countries.

In this same line, it was only possible to count on the perception of the students. However they are the main actors of the university system, it is advised to widen the scope and include the other actors of the educational community, so obtaining an integral vision that contains all viewpoints, as proposed in other studies around the world, both for schools and higher education [53, 55, 64, 66, 67].

## Conclusions

This study presents the Inclusive Management in Tertiary Institutions Scale (IMTIS) to assess the degree to which university management incorporates inclusive education guidelines.

The analyses carried out to find evidence about the validity and reliability of the scale measurements to assess inclusive management from the perspective of the student body, making it then possible to continue with the proposal of instruments that would allow the diagnosis of inclusive practices in the higher education level, as the evidence shows that these instruments are still scarce in this area [53–55].

To count on an instrument that assesses inclusive management from the viewpoint of the student body can contribute to carry out a constant diagnosis of the inclusion perceived by the students in the community. This is a key input for planning, management, and continuous supervision of university processes.

## Supporting information

S1 Table Dataset with students’ responses for the IMTIS scale. (XLS)
